# Prioritization of candidate genes in QTL regions based on associations between traits and biological processes

**DOI:** 10.1186/s12870-014-0330-3

**Published:** 2014-12-10

**Authors:** Joachim W Bargsten, Jan-Peter Nap, Gabino F Sanchez-Perez, Aalt DJ van Dijk

**Affiliations:** Applied Bioinformatics, Bioscience, Plant Sciences Group, Wageningen University and Research Centre, Wageningen, The Netherlands; Netherlands Bioinformatics Centre (NBIC), Nijmegen, The Netherlands; Laboratory of Bioinformatics, Plant Sciences Group, Wageningen University and Research Centre, Wageningen, The Netherlands; Laboratory for Plant Breeding, Plant Sciences Group, Wageningen University and Research Centre, Wageningen, The Netherlands; Biometris, Wageningen University and Research Centre, Wageningen, The Netherlands

**Keywords:** Quantitative trait locus, Candidate gene prioritization, Gene function prediction

## Abstract

**Background:**

Elucidation of genotype-to-phenotype relationships is a major challenge in biology. In plants, it is the basis for molecular breeding. Quantitative Trait Locus (QTL) mapping enables to link variation at the trait level to variation at the genomic level. However, QTL regions typically contain tens to hundreds of genes. In order to prioritize such candidate genes, we show that we can identify potentially causal genes for a trait based on overrepresentation of biological processes (gene functions) for the candidate genes in the QTL regions of that trait.

**Results:**

The prioritization method was applied to rice QTL data, using gene functions predicted on the basis of sequence- and expression-information. The average reduction of the number of genes was over ten-fold. Comparison with various types of experimental datasets (including QTL fine-mapping and Genome Wide Association Study results) indicated both statistical significance and biological relevance of the obtained connections between genes and traits. A detailed analysis of flowering time QTLs illustrates that genes with completely unknown function are likely to play a role in this important trait.

**Conclusions:**

Our approach can guide further experimentation and validation of causal genes for quantitative traits. This way it capitalizes on QTL data to uncover how individual genes influence trait variation.

**Electronic supplementary material:**

The online version of this article (doi:10.1186/s12870-014-0330-3) contains supplementary material, which is available to authorized users.

## Background

The elucidation of genotype-to-phenotype relationships remains a major challenge in biology. The causal relationship between variation of a trait-of-interest and genotypic differences is important for understanding genome evolution and functioning. In plants, it is the basis for developing targeted strategies in molecular breeding [1,2]. Technological developments in high-throughput phenotyping and next generation sequencing (NGS) are revolutionizing the scale of determination of phenotypes and genotypes [3,4].

A current bottleneck is the integration of all these data to unravel the molecular mechanisms behind traits-of-interest. Quantitative Trait Locus (QTL) mapping is an attractive approach to link genetic determinants to phenotypes [5-8]. In combination with physical maps, QTL studies have identified numerous genomic regions of various plants responsible for variation in particular traits. QTL analyses often are the primer to candidate gene mapping [9], but experimental approaches to identify the causal genes underlying a QTL are labor-intensive, time-consuming and expensive [10]. The limited number of crosses that can reasonably be performed leads to a low number of recombinations, which in turn means that QTLs are generally mapped with a low resolution: QTL regions typically contain tens to hundreds of genes.

Therefore, methods that help prioritizing QTL candidate genes using a computational approach would be very helpful in unraveling genotype-to-phenotype relationships. Such prioritization is well developed in human disease genetics, where several criteria, such as the putative deleteriousness of a variant, evolutionary conservation, and known biological pathways, are taken into account [11-23]. However, in plant biology and breeding, QTL candidate gene prioritization is much less developed. One approach consists of using genes previously identified as influencing the trait under study and test whether these explain a QTL [24,25], but this approach is limited to existing knowledge about genotype-to-phenotype relationships. Other approaches focus on integrating and visualizing existing information for prioritization [26-28] or merely give an overview of previously determined QTL candidate genes [29,30]. Little use has been made of biological pathways or predicted gene functions [31-33].

As an alternative experimental approach, genome-wide association studies (GWAS), which take advantage of historical recombination events, are able to increase resolution. However, GWAS can suffer from problems such as confounding due to genetic background, or diminishing power to find associations for rare alleles [5]. Moreover, existing diversity in a population available for GWAS analysis need not be relevant for a trait-of-interest.

**Figure 1:**
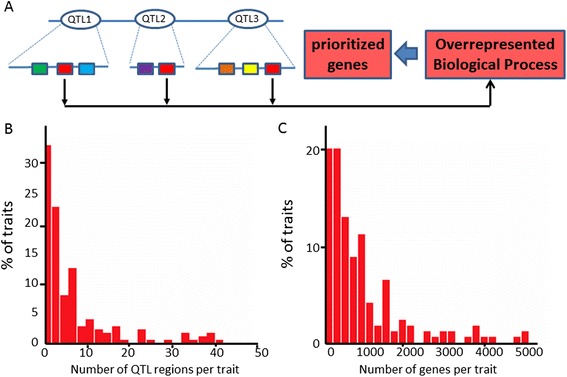
**Prioritizing QTL candidate genes via associating traits to biological processes. (A)** Principle of method used: Biological processes (indicated as different colored boxes) are annotated for genes in QTL regions for a trait-of-interest. Using these gene functions, trait-biological process associations are obtained based on enrichment of biological processes among the genes linked to a particular trait, integrating information from multiple QTL regions. Genes annotated with overrepresented biological processes are prioritized. **(B)** Number of QTL regions connected to traits in the rice QTL compendium used for this analysis. The scale of the horizontal axis in the histogram is clipped at 50, so traits with more than 50 QTL regions associated (~2% of the total) are not included. **(C)** Number of genes connected to traits in the rice QTL compendium. The scale of the horizontal axis in the histogram is clipped at 5000, so traits with more than 5000 genes associated (~5% of the total) are not included.

We here present a novel computational method for plant QTL candidate gene prioritization. In our approach (Figure 1A), for each gene contained in every QTL region for a trait-of-interest, we first predict which biological processes it is involved in. This is done using our previously developed gene function prediction method BMRF, which uses sequence data and co-expression information as input [34]. Enrichment (overrepresentation) of biological process (BP) terms, preferably based on multiple QTL regions for a given trait, allows association of the trait-of-interest with specific biological processes. Overrepresented BP terms are used to prioritize the candidate genes from the QTL gene lists that are most likely to be the underlying causal genes responsible for the variation in the trait-of-interest.

We applied this method in rice (*Oryza sativa*), chosen because of the large amount of QTL data available [35]. For a series of traits, we demonstrate the performance of candidate gene prioritization by comparing predictions with sets of genes known to be involved in the traits analyzed. On average, for 153 rice traits, a ten-fold reduction in the number of candidate genes was obtained by our prioritization. These results enable to capitalize on QTL data to uncover how individual genes influence trait variation.

## Methods

### From traits to genes

For 231 traits, QTL intervals reported as significant were extracted from the rice Gramene QTL compendium [35]. Genes in the QTL intervals were obtained from rice genome build 2009-01-MSU downloaded from Gramene [36]. To prevent too large regions to be used, a cutoff on maximum number of genes for a QTL interval was set to 450 genes; QTL regions containing more genes were excluded. This was based on testing the number of associations obtained for various size cutoffs (Additional file 1, SI Text).

### Linking genes to function

To predict gene functions (biological processes), BMRF [37-39] was applied using the PlaNet coexpression network [40] in combination with Argot2 [41] as recently described [34]. We compared the prioritization results obtained with these annotations with alternative existing function annotation from phytozome [42].

### Linking traits to function

For a set of genes contained in QTL regions associated with a particular trait, the occurrence of associated Gene Ontology BP terms was compared with the overall occurrence of these terms in the respective genome. To assess statistical significance, Fisher exact tests were applied as implemented in the R-function fisher.exact [43]. To adjust for multiple testing, a multiple testing correction was applied with the Benjamini-Hochberg method as implemented in the R-function p.adjust [44].

As part of the overrepresentation and gene prioritization analysis, three parameters were defined: (1) The False Discovery Rate (FDR) which defines the stringency of the multiple testing correction applied to the results of the Fisher exact test; (2) the minimum fraction of QTL regions for the trait-of-interest in which the BP term should at least occur; this prevents the use of statistically enriched BP terms present only in a small number of QTL regions; and (3) the maximum allowed BP term generality; i.e., only BP terms were used for which not too many genes were annotated genome-wide, to prevent the use of BP terms which are enriched in the QTL regions for a trait but which are very general and not likely to be useful for candidate gene prioritization. In order to find optimal values for these three parameters, the prioritized genes were compared with a set of known causal genes underlying QTLs (Additional file 1: Figure S1). The agreement between the prioritization predictions and the known causal genes was expressed as a p-value, based on comparison of the known causal QTL genes with randomly selected gene sets (see next section). Analyses presented in the paper used the optimized parameter values: FDR = 0.1, occurrence of the BP in at least 50% of the regions, and generality of the BP term not higher than 1%.

To compare the results of this procedure applied to an input set consisting of randomized gene function annotations, predicted gene functions were randomly reassigned to rice genes.

### Comparison with experimental datasets and analysis of prioritized candidate genes

Candidate genes occurring in QTL regions were prioritized based on their annotation with at least one of the overrepresented biological processes. To validate these predictions, a set of fine-mapped candidate genes was obtained from the literature. Identifiers of fine-mapped genes were either obtained directly from the publications in which they were reported, or converted using the information from RAP-DB (http://rapdblegacy.dna.affrc.go.jp/download/latest/RAP-MSU.txt.gz).

To assess the significance of fine-mapped gene retention after prioritizing genes, random gene sets were selected out of the QTL regions associated to the various traits; the size of these gene sets for each trait was identical to the number of genes selected by the prioritization approach. This was repeated 1,000 times, and to obtain a p-value, it was counted how many of the random folds retained at least the same number of fine-mapped genes as the number observed with the prioritization approach.

Comparison of prioritized candidate genes with transcription factors was performed using a list of rice transcription factors obtained from http://planttfdb.cbi.edu.cn/download/gene_model_family/Osj [45]. Comparison of predicted candidate genes with rice GWAS data was performed using data from two previous studies [8,46]. For each SNP reported as associated to a trait in those two studies, the three genes located closest to that SNP were considered as potentially causal candidates and were compared with the genes predicted based on QTL gene prioritization.

## Results

### QTL candidate gene prioritization

Our prioritization approach is based on the assumption that multiple QTL regions for a trait reflect variation in genes involved in the same biological process. To test this assumption, a dataset collected from various rice QTL mapping studies was used, as available in the Gramene database [35,36]. This set comprised in total 231 different traits, divided over nine different categories: abiotic stress, anatomy, biochemical, biotic stress, development, quality, sterility or fertility, vigor, and yield. Each of these traits was linked to one or more QTL regions, which were anchored along the rice genome. We removed from subsequent analyses each QTL region with more than 450 genes (Additional file 1, SI Text). Out of the 231 traits, the large majority (179, i.e. 77%) was associated with QTL regions that passed this size threshold, involving 1,591 QTL regions (Table 1). The distribution of the number of QTL regions per trait is presented in Figure 1B. Most traits (148 out of 179, i.e. 83%) are linked to multiple QTL regions; 68% of the traits (121) are linked to at least three QTL regions. This is important because as mentioned above, the prioritization approach is based on the assumption that multiple QTL regions for a trait reflect variation in genes involved in the same biological process. For all traits in the dataset, the associated genes were obtained from the genomic positions of the QTL regions. The average number of genes in a given QTL region is 140 ± 121 (± standard deviation). The number of genes per trait is given in Figure 1C; the total number of genes associated to each trait was on average 1,248 ± 1,869. In total, 38,366 genes were present in at least one QTL region; this is almost identical to the total number of genes in our rice functional annotation (38,998). Overall, these numbers clearly indicate the limited resolution of QTL data and emphasize the need for prioritization (See Table 2).

**Table 1:** **Associations between traits and biological processes** ^**a**^

**Input data**	
**#traits**	179
**#QTL regions**	1591
**#BP terms**	1767
**#relevant BP terms** ^**b**^	1522
**Prioritization results**	
**#trait-BP associations**	2519
**#traits involved**	153
**#BP terms involved**	918

^a^As intermediate step in candidate gene prioritization, traits and biological processes (BPs) were associated using overrepresentation of biological processes found for genes connected with each trait in the rice Gramene QTL compendium.

^b^Only BP terms which were associated with less than 1% of the genes in the genome were used as input terms in our analysis (i.e., a filter on the maximum allowed generality of the biological process was applied).

**Table 2:** **Candidate gene prioritization: comparison with QTL fine-mapping** ^**a**^

**Trait and fine-mapped candidate gene**	**#genes**	**#sel**	**Overrepresented biological processes involved**
Leaf size:	214	21	regulation of flower development
LOC_Os01g11940 [47]			lysine biosynthetic process via diaminopimelate
Leaf size:	214	21	organic acid catabolic process
LOC_Os01g11946 [47]			
Number of spikelets per panicle: LOC_Os01g12160 [48]	246	8	systemic acquired resistance
Gel consistency:	167	14	monosaccharide metabolic process
LOC_Os06g04200 [49]			glycolipid biosynthetic process
			membrane lipid biosynthetic process
			glucose metabolic process
Gelatinization temperature:	53	3	monosaccharide metabolic process
LOC_Os06g12450 [50]			glycolipid biosynthetic process
			membrane lipid biosynthetic process
Heading date:	330	13	positive regulation of RNA metabolic process
LOC_Os08g07740 [51]^b^			positive regulation of nucleobase-containing compound metabolic process
			positive regulation of (macromolecule/cellular) metabolic process
Yield, plant height:	188	8	positive regulation of macromolecule/cellular/nitrogen compound biosynthetic process
LOC_Os08g07740 [52]^b^			positive regulation of gene expression
Grain size and quality:	300	29	regulation of post-embryonic development
LOC_Os08g41940 [53]			
Viscosity parameter:	120	4	monosaccharide/glucose meta-/catabolic process
LOC_Os08g42410 [54]			glycolysis
			hexose catabolic process
			alcohol catabolic process

^a^For each trait found in literature with a fine-mapped candidate gene, QTL traits in our dataset were obtained which were similar/related to the literature trait, and for which the fine-mapped gene occurred in one of the QTL regions. Only cases for which the candidate gene was correctly prioritized by our approach are shown, in combination with the biological processes involved. #genes, number of genes in the input QTL region. #sel, total number of genes prioritized in the QTL region. For complete overview of comparison with fine-mapped candidate genes, see Additional file 3: Table S3.

^b^LOC_Os08g07740 is found as fine-mapped candidate gene for two different traits.

Associations between traits and biological process (BP) terms as defined in the Gene Ontology (GO) [55] were generated based on overrepresentation of BP terms in the QTL regions associated to a trait. As input BP terms we used our recently presented set of gene function predictions for rice [34], which consists of 1,767 different BP terms. On average, 23 BP terms occur per gene that can range from very high-level to very specific GO terms, and 494 ± 344 different BP terms occur in a QTL region. In order to focus only on BP terms which are not at a very high-level, a cutoff was applied on the maximum allowed number of genes annotated with a biological process genome-wide. In addition, a second cutoff was applied on the minimum fraction of QTL regions for a trait in which a BP should occur. The reasoning behind this cutoff was that a gene function reoccurring in multiple different QTL regions for the same trait is more relevant for candidate gene prioritization than a gene function that occurs several times in one QTL region for that trait. Values for these cutoffs are described in the Methods section and were obtained using comparison with genes fine-mapped as underlying QTLs.

For a given trait, we calculated overrepresentation of BP terms associated with all genes in all QTL regions (i.e. all candidate genes) as follows. From all candidate genes for the trait under investigation we determined the number of genes annotated with a particular BP term. This number was compared with the number of genes annotated with that same BP term in the whole genome. Enrichment was assessed using a Fisher exact test with multiple testing correction after testing for all traits and all biological processes. Within each QTL region for a given trait, genes associated with the overrepresented BP terms for that trait were identified as the candidate genes that are the most likely causal genes for that trait; we will refer to these as ‘prioritized candidate genes’. Because biological processes are intermediate in the process of candidate gene prioritization in this approach, we first discuss the biological processes selected, and then present the results of candidate gene prioritization based on these.

### Analysis of the association of traits with biological process terms

**Figure 2:**
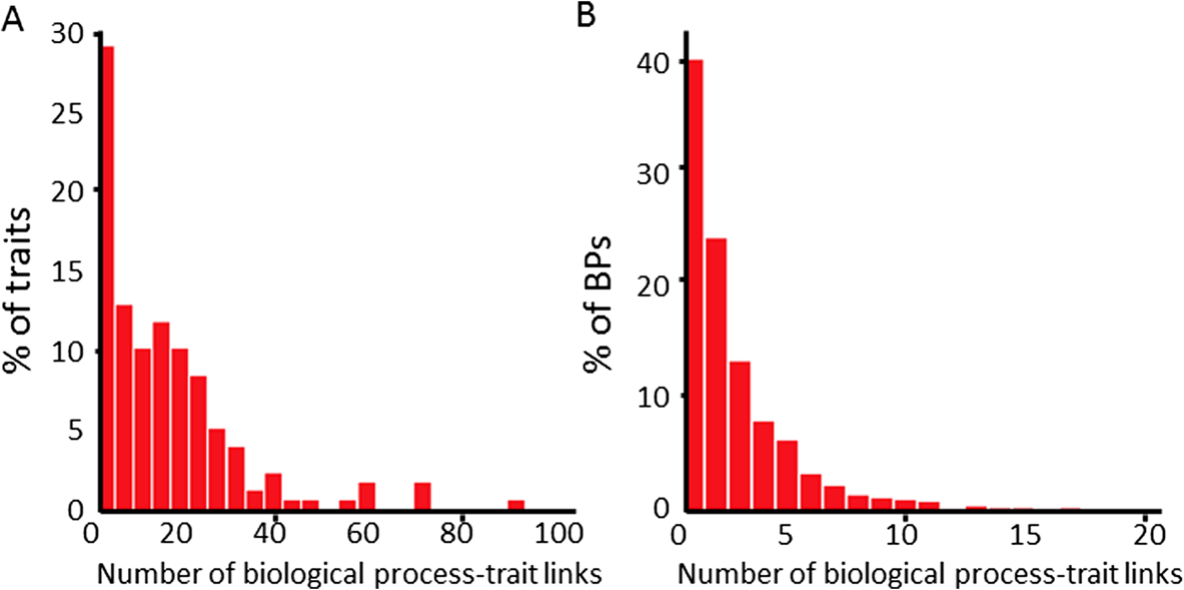
**Associations between traits and biological processes. (A)** Histogram of number of associations to biological processes (BPs) per trait. **(B)** Histogram of number of associations to traits per biological process.

From a list of 179 different traits in rice, for 153 traits 2519 associations with BP terms were obtained. For only 26 traits, no association with any BP was obtained at all. For most traits (134 out of 179, i.e. 75%) twenty or less BP term associations were obtained (Figure 2A). The detailed associations between traits and biological processes are given in (Additional file 2: Table S1) and summarized data are given in Table 1. In total, 918 BP terms (60%) were involved in at least one association to a trait (Figure 2B).

Inspection of these associations based on prior knowledge or through relevant literature shows that several connections were evident. These include the term ‘catabolic processes’ found for yield related traits; for the trait days to maturity, ‘carpel development’; for leaf height, ‘regulation of cell cycle process’; and for root activity both ‘organ development’ and ‘negative regulator of cell cycle’. Associations confirmed in literature include the link between the trait potassium uptake and glucose/galactose-related processes: potassium deficiency led to the inhibition of glycolysis and a build-up of root sugar levels in Arabidopsis [56]. For the yield trait ‘harvest index’ (weight of the harvested grain as percentage of total plant weight), the link with the BP ‘response to brassinosteroid stimulus’ is confirmed by the fact that manipulation of brassinosteroid level or brassinosteroid sensitivity influences yield [57].

To assess the significance of the obtained number of associations the procedure was repeated after randomly reassigning biological processes to genes. In this way no biological process-trait associations were obtained. In addition, we considered whether there is added value of using our BMRF function annotations for candidate gene prioritization compared to using alternative existing annotations. We found that existing rice gene function annotations resulted in less than half the number of associations obtained with our approach (data not shown). This confirms that our gene function annotation better enables to find associations between traits and BP terms. This is in line with the performance observed for our set of predictions, when comparing with experimentally determined gene functions [34]. This comparison indicated they were of high quality, demonstrating the added value of integrating sequence- and expression information for gene function prediction [34].

### Prioritization performance

The associations between traits and overrepresented biological processes allow narrowing down the number of candidate genes for a trait in a QTL region: genes associated with those BPs constitute the potentially causal genes. In total, for 153 traits, 6,175 prioritized candidate genes were obtained (Additional file 2: Table S2; see also www.ab.wur.nl/bmrftrait which allows to search on gene or trait), involving 1,120 different QTL regions. This involved a more than ten-fold reduction in the number of candidate genes: averaged over the traits, 9% ± 5% of QTL candidate genes were prioritized. Per QTL region, the average number of prioritized genes was 13 ± 13 which is indeed an over ten-fold reduction compared to the above-mentioned number of 140 ± 121 candidate genes per input QTL region. We assessed the relevance of the prioritization in several ways.

First, a simulation analysis indicated that overrepresented biological processes allow to preferentially select, i.e. prioritize, relevant candidate genes. Upon randomly adding genes to the set of genes present in the QTL regions for a trait, the enrichment analysis tends to identify genes that occur in the original QTL regions and not randomly added genes (Additional file 1). This shows that our prioritization protocol can do away with deliberately added noise.

Second, we compared the prioritization results with a set of genes in rice that were experimentally validated by QTL fine-mapping as truly causal gene for the trait-of-interest. To do so, fine-mapping results for various traits obtained from literature were matched to traits in the Gramene QTL database. This established a test set of 16 genes that should be prioritized in the analysis. Of these 16 genes, 8 were indeed prioritized by our approach (Table 2, Additional file 3: Table S3). The percentage of correctly prioritized candidate genes (8/16, 50%) is much higher than the above mentioned percentage of genes that is prioritized using our approach (9%). Hence, prioritization based on BP term overrepresentation reduces the number of candidate genes over tenfold while at the same time the loss of validated causal genes is only twofold. Compared with randomly selected gene sets, this is very significant (p < 0.001). Note that the set of fine-mapped causal genes used in this comparison was also used in setting the two cutoff values applied in our prioritization method (see above). Hence, this dataset does not constitute independent validation of our method. However, irrespective of the exact cutoff values chosen, prioritization results were always significant, except for a very high value of the cutoff on the fraction of QTL regions in which a prioritized BP should occur (>90%; Additional file 1: Figure S1). Changing the values of the applied cutoffs would allow to recover more truly causal genes, but at the expense of also obtaining a larger set of prioritized candidate genes overall. For example, when the cutoff on the maximum allowed percentage of genes annotated with a biological process genome-wide would be set to 20% instead of the chosen value of 1%, we would recover 13 out of 16 genes (80%) instead of 8 out of 16 (50%). However, with this setting, the average percentage of prioritized genes would be 25% (instead of 9%).

Note that uncertainty in the set of causal genes that we use as reference set will lead to an underestimate of the performance of our method in correctly prioritizing fine-mapped genes. There are at least three sources of such uncertainty. First, traits mentioned in the literature for which fine-mapped genes were found, were matched to traits in the rice QTL compendium available. However, in most cases, the trait was not exactly the same trait as the one for which fine-mapping was performed (Additional file 3: Table S3). In such cases, the causal gene underlying the literature trait might be different from the causal gene for the trait included in this analysis. Second, even when the trait is identical, the populations in the dataset and in the experimental study in which the candidate gene was fine-mapped do not need to be the same. The causal gene that was fine-mapped may therefore not be the causal gene in the QTL region we used. Third, available fine-mapping results do not always exclude that a neighboring gene is the actual causal gene. The resolution of fine-mapping is limited and often the causal gene is chosen from a small number of fine-mapped candidates based on e.g. molecular function. One example of both the first and third source of uncertainty is given by the gene *LOC_Os06g04820* fine mapped for the trait ‘small panicle and dwarfness’ [58]. This trait did not match exactly to a trait in our input set, but we used ‘plant height’ and ‘grain yield per plant’ as substitute traits, because some of the input QTL regions for those traits overlapped with the region analyzed in this reference. Our prioritization approach did not return *LOC_Os06g04820*. In addition to the potential mismatch between the traits, this could also be due to the fact that the fine-mapping by [58] did not identify *LOC_Os06g04820* unambiguously, but identified a group of four genes (*LOC_Os06g04810*, *LOC_Os06g04820*, *LOC_Os06g04830* and *LOC_Os06g04840*) among which LOC_Os06g04820 was chosen as the most likely candidate. Although neither of those other three genes was identified by our prioritization approach, a gene immediately neighbouring these genes, *LOC_Os06g04800*, was prioritized for both the traits ‘plant height’ and ‘grain yield per plant’ by our approach.

### Comparison with large scale experimental datasets

Further comparison with experimental data was performed using two large scale datasets. First, data from a rice database defining associations between in total 637 traits and 239 genes [59] were used. Most of these associations are not based on QTL fine-mapping but on e.g. analysis of mutants. This means that we do not necessarily expect a perfect agreement between those data and our predictions. For 26 gene-trait associations from this database both trait and gene were present in the QTL data, meaning that they could be used for this analysis. From these 26 cases, 8 gene-trait associations were identified (Table 3). This number is significant (p ~ 0.04), based on comparison with randomized gene-trait associations. Importantly, our results do not just recapitulate those experimentally known associations between traits and genes, but indicate which biological processes (gene functions) could be involved in those associations. Some of these biological processes (Table 3) are quite obvious (e.g. NADPH regeneration in relation to the trait chlorophyll content) but others give insight into complex traits such as plant height. For the latter, overrepresented biological processes include phosphorylation related processes, ethylene related processes, and processes related to pattern formation.

**Table 3:** **Validated causal genes** ^**a**^

**Gene**	**Trait**	**Overrepresented biological processes involved**
LOC_Os01g10840	plant height	intracellular protein kinase cascade; pattern specification process; xylem and phloem pattern formation; signal transduction by phosphorylation
LOC_Os01g58420	spikelet number	cellular response to ethylene stimulus
LOC_Os01g66120	plant height	positive regulation of macromolecule biosynthetic process/nitrogen compound metabolic process/gene expression
LOC_Os02g43790	spikelet number	cellular response to ethylene stimulus
LOC_Os03g03370	relative water content	microgametogenesis
LOC_Os08g06380	plant height	two-component signal transduction system (phosphorelay); ethylene mediated signaling pathway; cellular response to ethylene stimulus
LOC_Os09g26400	chlorophyll content	NADPH regeneration; nicotinamide nucleotide metabolic process
LOC_Os11g08210	plant height	positive regulation of macromolecule biosynthetic process/nitrogen compound metabolic process/gene expression

^a^Genes prioritized for traits based on overrepresentation of biological processes in QTL regions for the trait for which validation is available based on literature results [59].

Second, we screened the prioritization with the results of two rice GWAS studies [8,46]. For 14 traits in the Gramene QTL compendium, an equivalent trait was present in the GWAS data (Additional file 3: Table S4). For 12 of these traits, genes in QTL regions were prioritized. For these genes we assessed whether they were found in the neighborhood of significant SNPs identified by GWAS (neighborhood was defined as the three genes nearest to the GWAS SNP). Note that, similar as for the above presented comparison with gene-trait combinations, we do not expect perfect agreement between our QTL-based prioritization and the results of these GWAS studies. Nevertheless, 37 of the prioritized candidate genes were in the neighborhood of significant SNPs identified by GWAS; these involved 6 of the 12 traits. Comparison with randomized sets of genes selected from the QTL regions for those traits indicates that the number of 37 genes was significant (p ~ 0.03). Taken together, these results demonstrate that our prioritization strategy results in lists of prioritized candidate genes that are significantly enriched for trait-relevant genes.

### Importance of transcription factors among prioritized genes

An important question with respect to the prioritized candidate genes is whether these have any special properties which make them *a priori* more likely to be causal genes. In particular, we analyzed the role of transcription factors (TFs) among the prioritized candidate genes. In the rice genome, 3.1% of the genes are transcription factors [45], and in the set of all genes in the QTL regions (i.e. all candidate genes) it is 3.8%. However, in the set of prioritized candidate genes, the percentage of TFs is 11.0%. When distinguishing prioritized candidate genes associated to only one trait (2,758 in total) and those associated with more than one trait (3,417 in total), the percentage of TFs is higher in the latter: 13% for genes linked to at least two traits, and 15% for genes linked to at least four traits. The preference for TFs to be associated with traits is in line with the fact that in our input set of gene function predictions for rice, TFs obtain approximately twofold higher number of associated biological processes compared to other genes (not shown). This important role of TFs could explain the fact that QTLs associate preferentially with large-effect mutations [60].

**Figure 3:**
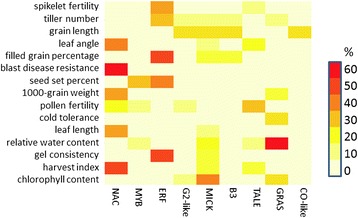
**Transcription factors as potentially causal genes.** Specific TF families (horizontal axis) were found associated with specific traits (vertical axis). Heatmap shows which percentage of the associated TFs belongs to various TF subfamilies for traits with at least ten associated TFs, and at least one TF subfamily which constitutes more than 25% of all associated TFs for that trait. Only TF subfamilies which for at least one trait constituted more than 25% of all TFs, are shown.

In addition to the overall higher number of transcription factors among the prioritized candidate genes, there are also clearly different types of transcription factors associated with specific traits (Figure 3). For several of these associations evidence exists in the literature. For example, the trait chlorophyll content is associated by our analysis with MICK MADS domain transcription factors; this is in line with the fact that targets of the tomato MADS TF RIN are involved in chlorophyll degradation [61]. The traits blast disease resistance and leaf angle are associated with NAC transcription factors by our analysis; experimental evidence indicates that these TFs are indeed involved in pathogen responses [62] and in waterlogging-induced upward bending of leaves [63]. Finally, the trait tiller number is associated with ERF transcription factors, and indeed the rice ERF TF OsEATB is known to be involved in regulation of tillering [64]. This preference of particular types of TFs to be relevant for specific traits will be useful in further prioritization of candidate genes for such traits.

### Example: analysis of QTL regions for the trait days to heading

**Figure 4:**
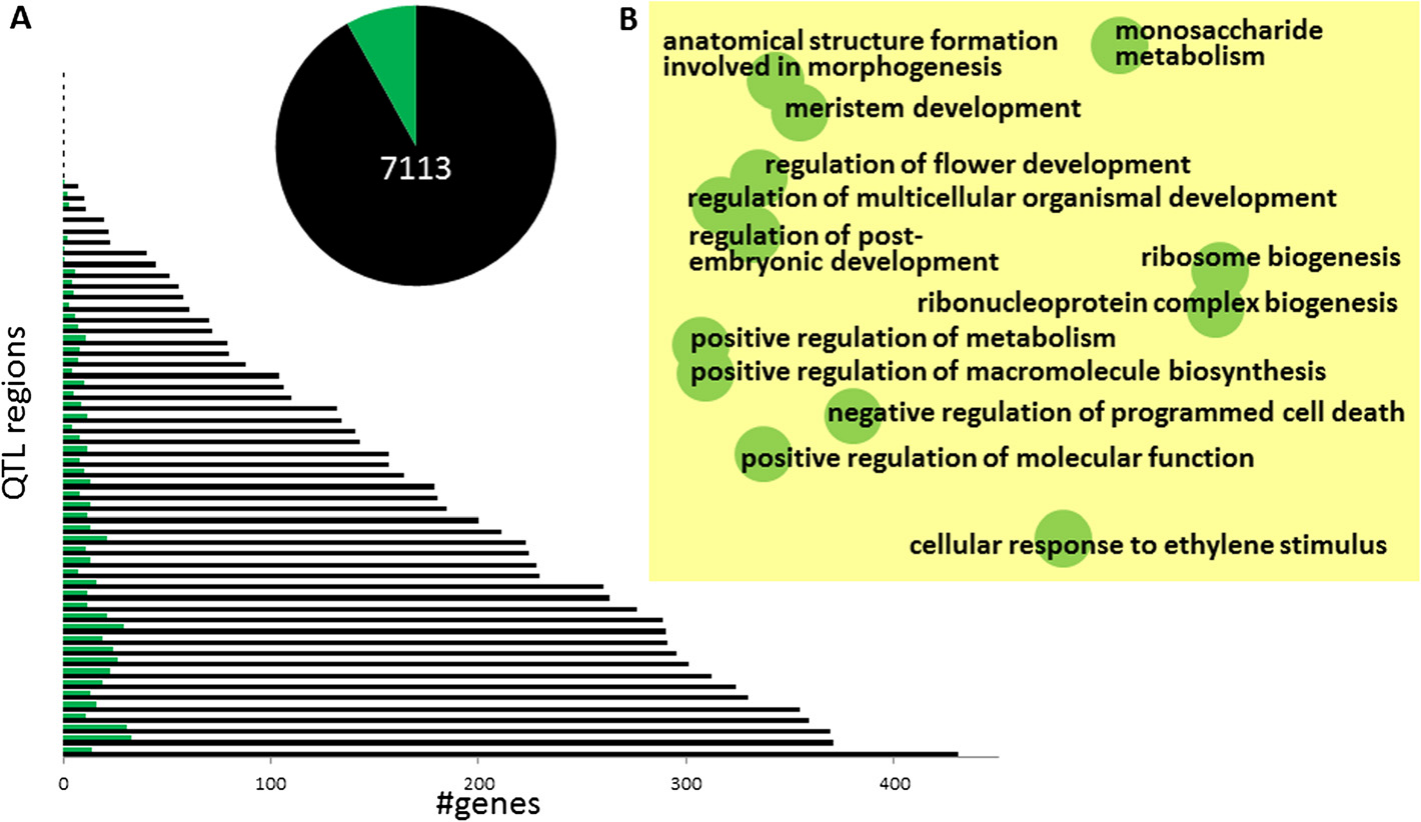
**Analysis of QTL regions for rice trait days to heading. (A)** Overview of prioritization results per QTL region. Each pair of horizontal bars indicates a QTL region; the black bar represents the total number of genes in the region, and the green bar the number of prioritized (potentially causal) genes. Inset: pie-diagram indicates the total number of genes (7113), and the fraction of those genes selected by the prioritization approach (579). **(B)** Overview of selected biological processes: REVIGO [68] scatterplot view in which each circle represents a BP; the distance between circles indicates similarity between BPs.

To illustrate the added value for plant biology, we considered the trait days to heading in depth. Days to heading, which is related to the trait flowering time, is an important parameter for rice breeding [65,66] and plays a key role in adaptation of rice to different environments [67]. In Figure 4A the number of genes prioritized is plotted, either divided per QTL region (main) or in all QTL regions together (insert). The various terms obtained for this trait are depicted in Figure 4B. Here, the position of each biological process term is chosen to represent similarities between the terms [68]. The overrepresented biological process occurring for the largest number of genes for this trait is ‘regulation of multicellular organismal development’. This term, although quite general, is obviously relevant for days to heading. Another relevant selected term was ‘cellular response to ethylene stimulus’; an ethylene receptor is known to delay the floral transition in rice [69]. A third clearly relevant term was ‘regulation of flower development’. We analyzed the genes associated with this term in more detail. From 7,113 genes in the rice QTL regions linked with the trait days to heading, 79 genes were assigned to the term ‘regulation of flower development’ by our function annotation (Additional file 3: Table S5) and hence prioritized as potentially causal genes for this trait by our method. Of these 79 genes some are described as ‘unknown’ by existing annotations (Additional file 3: Table S5). For example, gene *LOC_Os04g54420* is annotated as containing a domain of unknown function (DUF618). Such genes could not have been prioritized based on existing annotations, which illustrates the importance of using our set of computational gene function predictions as input. To have a closer look at the genes prioritized for the trait days to heading based on the BP ‘regulation of flower development’ we focused on the genes that in the QTL region in which they occur were the only gene associated with this BP. Given the relevance of the BP ‘regulation of flower development’ for the trait days to heading, the occurrence of only one gene annotated with that BP term in a QTL region for this trait makes that gene a prime candidate for further study. There are in total 11 of such genes (Table 4). Analysis of the existing Rice Genome Annotation Project data [70] for these genes indicates that some are known to be involved in flower development. This includes two MADS genes, *OsMADS34*, involved in inflorescence and spikelet formation [71], and *OsMADS18*, involved in specifying floral determinacy and organ identity [72]. Several other genes are however not characterized at all and should therefore be considered new potentially causal genes involved in the regulation of flowering time. This includes a MYB transcription factor and two zinc finger domain containing proteins. In line with the preference for TFs among prioritized candidate genes, the set of 11 genes contains 5 TFs: the three mentioned above (2x MADS, 1x MYB) as well as two GATA TFs.

**Table 4:** **Genes predicted as causal genes for days to heading** ^**a**^

**Gene**	**Available existing annotation**
LOC_Os01g68620	signal peptide peptidase-like 2B
LOC_Os01g70920	cullin-1
LOC_Os01g74020	MYB family transcription factor
LOC_Os03g54170	OsMADS34 - MADS-box family gene with MIKCc type-box
LOC_Os03g61570	expressed protein
LOC_Os05g02300	Core histone H2A/H2B/H3/H4 domain containing protein
LOC_Os07g41370	OsMADS18 - MADS-box family gene with MIKCc type-box
LOC_Os07g46180	PWWP domain containing protein
LOC_Os07g08880	ES43 protein
LOC_Os09g39270	ZOS9-20 - C2H2 zinc finger protein
LOC_Os10g40810	GATA zinc finger domain containing protein

^a^Genes prioritized in QTL regions for trait days to heading based on their predicted function ‘regulation of flower development’, and present as single gene annotated with this term in the respective QTL region. Without the last requirement, in total 79 genes were prioritized in the QTL regions for this trait based on the BP ‘regulation of flower development’ (Additional file 3: Table S5).

Among the biological processes associated with the trait days to heading, the related processes ‘ribonucleoprotein complex biogenesis’ and ‘ribosome biogenesis’ had only low similarity to other biological processes associated with this trait; this is indicated by their position relative to other terms in Figure 4B. In total, 72 genes involved in these two biological processes are prioritized as potentially causal genes for days to heading (Additional file 3: Table S6). Although a role of the ribosome in flowering time has not been described in great detail, circumstantial evidence in the literature suggests that the ribosome might indeed be important. In particular, TOR kinase which mediates ribosomal biogenesis, regulates flowering and senescence in Arabidopsis [73]. In maize, a protein involved in translation initiation has been confirmed as underlying a flowering time QTL [74], and in *Solanum chacoense*, a protein involved in ribosome biogenesis influenced flowering [75].

These examples show how the approach taken to link traits with biological processes and subsequently to genes can generate relevant leads for future laboratory experimentation.

## Discussion

In order to exploit the information hidden in plant genomics data for breeding, better understanding of genotype-to-phenotype relationships is essential. The biological and molecular basis of most quantitative trait variation is poorly understood and QTL mapping approaches generally result in too large numbers of candidate genes to be able to identify causal genes easily. The prioritization of candidate genes is not only of fundamental interest, but also of high practical value, because causal genes for any trait-of-interest make perfect markers for breeding. Our results demonstrate that associations between overrepresented biological processes and traits help to prioritize candidate genes and zoom in on the potentially causal genes for the trait-of-interest. Our integrated analysis is the first large-scale application assessing explicitly the performance of overrepresentation of predicted gene functions for the identification of potentially causal genes for plant traits in genomic regions obtained by QTL mapping.

Our approach resulted in a reduction in total number of genes of more than ten-fold compared to the number of genes in the input QTL regions. Based on comparison with different experimental datasets, the predicted causal genes are clearly statistically significant. Although we could only compare the prioritized genes with a limited number of fine-mapped genes available in literature, our predictions enable to test potentially causal genes underlying QTLs at a larger scale. This paves the way towards obtaining more detailed insight into the role of specific genes underlying QTLs which in turn should enable further validation of our predictions in the future. As demonstrated by the example of genes prioritized for days to heading, included in the set of prioritized genes are genes with so far completely unknown function. Such genes will be particularly interesting targets for experimental verification.

Out of 179 traits, for 26 no predictions were obtained. It could be that for some of these 26 traits, causal genes underlying different QTL regions are not involved in the same biological process. If indeed for each QTL region for a trait-of-interest a different biological process would be underlying, our enrichment analysis would not be able to predict these biological processes. However, for ~30% (8 of 26) of these traits only one QTL region was available, two times the percentage of traits with only one QTL region observed overall (~15%). This indicates that traits with multiple QTL regions are more likely to indeed contain overrepresented BPs. In other words, the analysis of overrepresented BPs profits from the availability of multiple QTL regions. This is in line with the above mentioned assumption underlying our prioritization method, that multiple QTL regions for a trait reflect variation in genes involved in the same biological process. Taken together, our results clearly indicate that this assumption is often correct.

We found that transcription factors are prominently present among the prioritized candidate genes. This points towards an explanation for the fact that QTL studies preferably find large effect mutations [60]. It may also emphasize the important role of transcription factors in domestication. Half to two-third of genes known to be involved in domestication consist of transcription factors [76,77] and many of the traits important for breeding are relevant in the context of domestication [78].

The input needed for prioritization as here developed consists of QTL regions and predicted gene functions. Incorporating the significance level of the association of genome regions with a trait using QTL Logarithm Of the Odds (LOD) scores could improve the analysis as could better assessment of the overrepresentation of biological process terms using e.g. gene set enrichment analysis [79], iterative group analysis [80], or approaches that take the hierarchy of the Gene Ontology into account [81]. Yet, in such enrichment analysis the importance of the source of the gene function annotations is often underestimated. Especially in case of agricultural crops, knowledge of what all the genes predicted to be present in the genome are actually doing, is scarce [82]. For example, existing databases describing rice gene functions only contain relatively small number of cases [59,83]. Having a large set of high-quality gene function predictions [34] results in much higher numbers of significant associations between traits and biological processes compared to using existing annotations.

## Conclusions

The set of potentially causal genes that results from the prioritization approach here demonstrated could be an important dataset for future applications in rice breeding. Other crops as well as relevant animal species could be addressed in a similar way. It may motivate research communities to generate the data necessary for such analyses. QTL data are available for various plant species and we generated sets of high-quality biological process predictions for different plant species, including major crops [34]. In the future it should be possible to analyze data from various species simultaneously to find overrepresented biological processes among QTL regions linked to the same trait in different species. Such comparative approach will help to extract more useful information from available data in order to elucidate and exploit the link between genotype and phenotype.

## Additional files

Additional file 1:
**Supplementary Text and Figure S1.**
Additional file 2:
**Supplementary Tables I and II.** **Table S1** contains associations between traits and biological processes. **Table S2** contains prioritized candidate genes and their associated traits. This information is also available via www.ab.wur.nl/bmrftrait. Additional file 3:
**Supplementary Tables III-VI.** **Table S3** contains comparison with fine-mapping results; **Table S4** comparison with GWAS results. **Table S5** lists genes in QTL regions for “heading date” annotated with “regulation of flower development”. **Table S6** lists genes in QTL regions for “heading date” annotated with “ribonucleoprotein complex biogenesis” and “ribosome biogenesis”.
