# Supplementary material for: Prioritization of candidate genes in QTL regions based on associations between traits and biological processes
Source: BMC Plant Biol. 2014 Dec 10;14:330. doi: 10.1186/s12870-014-0330-3 (PMC4274756; doi:10.1186/s12870-014-0330-3)
Supplement: Additional file 1: — Supplementary Text and Figure S1. [file 12870_2014_330_MOESM1_ESM.doc]

**Prioritization of candidate genes in QTL regions based on associations between traits and biological processes**

Joachim W. Bargsten^1,2,4^, Jan-Peter Nap^1,2^, Gabino F. Sanchez-Perez^1,3^, Aalt D. J. van Dijk^1,5^*

^1^ Applied Bioinformatics, Bioscience, Plant Sciences Group, Wageningen University and Research Centre, Wageningen, The Netherlands

^2^ Netherlands Bioinformatics Centre (NBIC), Nijmegen, The Netherlands

^3^ Laboratory of Bioinformatics, Plant Sciences Group, Wageningen University and Research Centre, Wageningen, The Netherlands

^4^ Laboratory for Plant Breeding, Plant Sciences Group, Wageningen University and Research Centre, The Netherlands

^5^ Biometris, Wageningen University and Research Centre, Wageningen, The Netherlands

* Corresponding author: aaltjan.vandijk@wur.nl

**Supporting Information Text**

**QTL regions size threshold**

An important parameter to consider is the maximum size of QTL regions that are included in the analyses. Only using small regions could filter out intervals with useful information, whereas using too large regions could add too much noise (irrelevant genes). Based on *in silico* experimentation we concluded that the QTL region should not be larger than 450 genes, because this resulted in the largest number of statistically significant associations between traits and biological processes (data not shown). Out of the total of 1591 QTL regions passing this threshold, 1066 contain at least 50 genes, and 896 contain at least 100 genes. However, a subset of the regions is small: there are 350 regions with less than 5 genes, and most of these (327 regions) contain only one gene. The latter is indicative of “single point QTL analysis”, in which only the marker with highest correlation to the trait was reported and stored in the database, and not a complete QTL region was reported . We tested the influence of these very small QTL regions by performing an analysis in which an additional cutoff on the minimum number of genes that should be present in a QTL region was applied. This showed that the results were not much influenced by this additional cutoff. When requiring that at least 100 genes should be present in a QTL region, the number of predicted associations was only roughly 10% lower than when using all regions. Moreover, only around 10% of the BP – trait associations obtained when only using regions with at least 100 genes, were not found when using all regions. Given the relatively small impact all analyses presented in the main text included the upper limit criterion only.

**Relevance of trait-biological process associations for obtaining causal genes: simulation**

We tested the performance of the predicted trait-biological process link for prioritizing candidate genes using a simulation approach. For each trait in this set, we added randomly chosen genes to the set of genes linked to it, and then reasoned that our analysis should preferably find biological processes linked with genes in the original set of genes. When adding either 10% or 50% additional genes, the biological processes that were predicted as linked to the traits were to a large extent a subset of those that were obtained with the original data (not shown). Of importance in the context of candidate gene prioritization, genes that were linked with the overrepresented biological processes in the enlarged datasets preferentially occurred in the original input set, an effect that was more pronounced for GO terms that were less general. For example, when adding 10% (50%) extra genes, random expectation would be that 100/110=91% (100/150=67%) of the selected genes would be in the original input region, but the overall percentages were 97%+/-8% (85%+/-19%). This is a clear improvement over random expectation. In addition, there was a strong negative correlation (Spearman rho ~ -0.35; p-value < 10^-16^) between this percentage for various biological processes and the generality of these GO terms (percentage of genes predicted for that process), meaning that for less general biological processes the effect was even stronger.

**Supporting Information Figure**


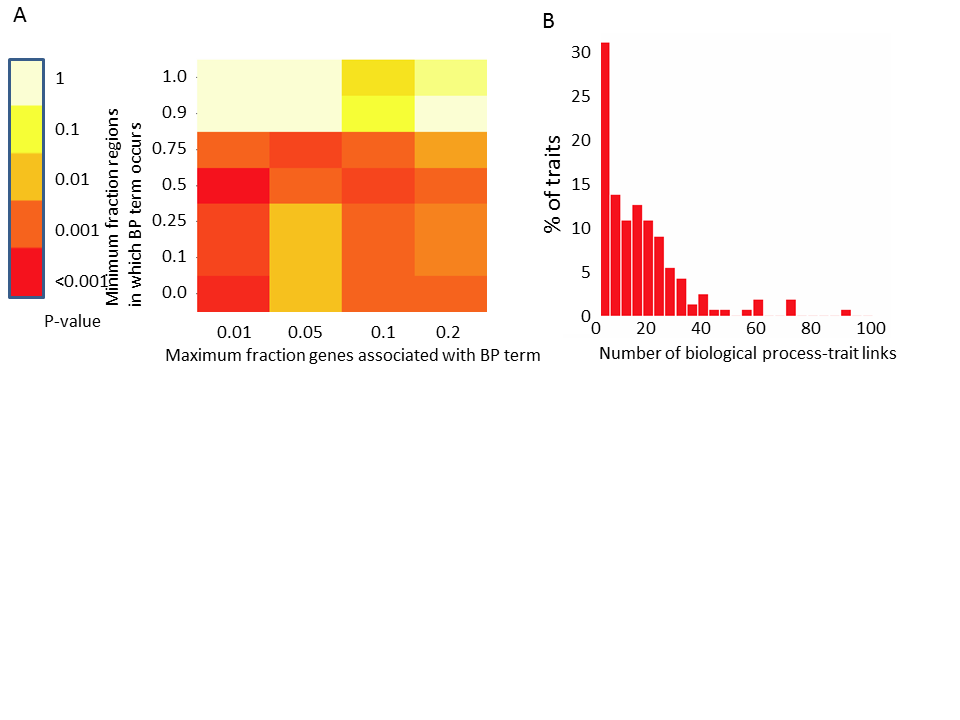


**Figure S1.** Dependence of candidate gene recovery performance on cutoff on fraction of genes associated with BP term, and on fraction of regions in which BP term should occur. P-value is estimated by assessing in how many of 1,000 random sets of selected genes at least the same number of validated candidate genes is recovered as with the prioritization approach.
