# Supplementary material for: Prioritization of candidate genes in QTL regions based on associations between traits and biological processes
Source: BMC Plant Biol. 2014 Dec 10;14:330. doi: 10.1186/s12870-014-0330-3 (PMC4274756; doi:10.1186/s12870-014-0330-3)
Supplement: Additional file 3: — Supplementary Tables III-VI. Table S3 contains comparison with fine-mapping results; Table S4 comparison with GWAS results. Table S5 lists genes in QTL regions for “heading date” annotated with “regulation of flower development”. Table S6 lists genes in QTL regions for “heading date” annotated with “ribonucleoprotein complex biogenesis” and “ribosome biogenesis”. [file 12870_2014_330_MOESM3_ESM.doc]

**Prioritization of candidate genes in QTL regions based on associations between traits and biological processes**

Joachim W. Bargsten^1,2,4^, Jan-Peter Nap^1,2^, Gabino F. Sanchez-Perez^1,3^, Aalt D. J. van Dijk^1,5^*

^1^ Applied Bioinformatics, Bioscience, Plant Sciences Group, Wageningen University and Research Centre, Wageningen, The Netherlands

^2^ Netherlands Bioinformatics Centre (NBIC), Nijmegen, The Netherlands

^3^ Laboratory of Bioinformatics, Plant Sciences Group, Wageningen University and Research Centre, Wageningen, The Netherlands

^4^ Laboratory for Plant Breeding, Plant Sciences Group, Wageningen University and Research Centre, The Netherlands

^5^ Biometris, Wageningen University and Research Centre, Wageningen, The Netherlands

* Corresponding author: aaltjan.vandijk@wur.nl

**Table S3. Comparison with fine-mapping results^a^**

| ***Fine-mapped gene*/**  **QTL compendium trait** | | ***Literature trait*/ BP** | | | | | | | **BP Name** | | | |  |  | | |  |
| --- | --- | --- | --- | --- | --- | --- | --- | --- | --- | --- | --- | --- | --- | --- | --- | --- | --- |
| *LOC_Os01g10110* | | *Grain number* | | |  | | | | | | | |  |  | | |  |
| Grain yield | | | NA | | | | |  | | | |  | | | | | |
| Filled grain number | | | NA | | | | |  | | | |  | | | | | |
| *LOC_Os01g11940* | | *Leaf size* | | |  | | | | | | | |  |  | | |  |
| Leaf senescence | | | GO:0009909 | | | | | | regulation of flower development | | | | | | |  | |
| Leaf senescence | | | GO:0051093 | | | | | | lysine biosynthetic process via diaminopimelate | | | | | | |  | |
| Large vascular bundle number | | | NA | | | | | |  | | | | | | |  | |
| *LOC_Os01g11946* | | *Leaf size* | | |  | | | | | | | |  |  | | |  |
| Leaf senescence | | | GO:0016054 | | | | | | organic acid catabolic process | | | | | | | | |
| Large vascular bundle number | | | NA | | | | | |  | | | | | | | | |
| *LOC_Os01g12160* | | *Number of spikelets per panicle* | | | | | | | | | | | |  | | |  |
| Panicle weight | | | NA | | | | | |  | | | | | | | | |
| Spikelet number | | | GO:0009627 | | | | | | systemic acquired resistance | | | | | | | | |
| *LOC_Os03g63970* | | *Seedling vigor, plant height* | | | | | | | | | | | |  | | |  |
| Root activity | | | NA | | | | | |  | | | | | | |  | |
| *LOC_Os04g33740* | | *Grain weight, grain filling* | | | | | | | | | | | | | | |  |
| Grain number | | | NA | | | | | | |  |  | | | | | | |
| Filled grain number | | | NA | | | | | | |  |  | | | | | | |
| *LOC_Os06g04200* | | *Gel consistency* | | |  | | | | | | | |  |  | | |  |
| Chalkiness of endosperm | | | GO:0005996 | | | | | | monosaccharide metabolic process | | | | | | | | |
| Chalkiness of endosperm | | | GO:0009247 | | | | | | glycolipid biosynthetic process | | | | | | | | |
| Chalkiness of endosperm | | | GO:0046467 | | | | | | membrane lipid biosynthetic process | | | | | | | | |
| Amylose content | | | NA | | | | | |  | | | | | | | | |
| Gelatinization temperature | | | GO:0005996 | | | | | | monosaccharide metabolic process | | | | | | | | |
| Gelatinization temperature | | | GO:0006006 | | | | | | glucose metabolic process | | | | | | | | |
| *LOC_Os06g04820* | | *Small panicle and dwarfness* | | | | | | | | | | | | | | |  |
| Plant height | | | NA | | | |  | | | |  | | | | | | |
| Grain yield per plant | | | NA | | | |  | | | |  | | | | | | |
| *LOC_Os06g12450* | | *Gelatinization Temperature* | | | | | | | | | | | | | | |  |
| Chalkiness of endosperm | | | GO:0005996 | | | | | | monosaccharide metabolic process | | | | | | |  | |
| Chalkiness of endosperm | | | GO:0009247 | | | | | | glycolipid biosynthetic process | | | | | | |  | |
| Chalkiness of endosperm | | | GO:0046467 | | | | | | membrane lipid biosynthetic process | | | | | | |  | |
| *LOC_Os06g45460* | | *Lodging resistance and yield* | | | | | | | | | | | | | | |  |
| Panicle number | | | NA | | |  | | | | | | | | | |  | |
| 1000-seed weight | | | NA | | |  | | | | | | | | | |  | |
| Spikelet number | | | NA | | |  | | | | | | | | | |  | |
| *LOC_Os08g07740* | | *Heading date* | | |  | | | | | | | |  |  | | |  |
| Days to heading | GO:0009893 | | | positive regulation of metabolic process | | | | | | | | | | |  | | |
| Days to heading | GO:0010557 | | | positive regulation of macromolecule biosynthetic process | | | | | | | | | | |  | | |
| Days to heading | GO:0010604 | | | positive regulation of macromolecule metabolic process | | | | | | | | | | |  | | |
| Days to heading | GO:0031325 | | | positive regulation of cellular metabolic process | | | | | | | | | | |  | | |
| Days to heading | GO:0045935 | | | positive regulation of nucleobase-containing compound metabolic process | | | | | | | | | | |  | | |
| Days to heading | GO:0051254 | | | positive regulation of RNA metabolic process | | | | | | | | | | |  | | |
| *LOC_Os08g07740* | | *Yield, plant height* | | | | | |  | | | | | | | | |  |
| Plant height | GO:0010557 | | | positive regulation of macromolecule biosynthetic process | | | | | | | | | | |  | | |
| Plant height | GO:0010628 | | | positive regulation of gene expression | | | | | | | | | | |  | | |
| Plant height | GO:0051173 | | | positive regulation of nitrogen compound metabolic process | | | | | | | | | | |  | | |
| 1000-grain weight | GO:0031325 | | | positive regulation of cellular metabolic process | | | | | | | | | | |  | | |
| 1000-grain weight | GO:0051173 | | | positive regulation of nitrogen compound metabolic process | | | | | | | | | | |  | | |
| Filled grain number | NA | | |  | | | | | | |  | | | | | | |
| *LOC_Os08g41940* | | *Grain size, width, shape and quality* | | | | | | | | | | | |  | | |  |
| Filled grain percentage | | | GO:0048580 | | | | | | regulation of post-embryonic development | | | | | | | | |
| Yield per plant | | | NA | | | | | |  | | | | | | | | |
| *LOC_ Os08g42268* | | *Viscosity parameter (related to eating quality)* | | | | | | | | | | | | | | |  |
| Soluble protein content | | | NA | | | |  | | | |  | | | | | | |
| Gelatinization temperature | | | NA | | | |  | | | |  | | | | | | |
| *LOC_Os08g42410* | | *Viscosity parameter (related to eating quality)* | | | | | | | | | | | | | | |  |
| Soluble protein content | | | NA | | | | | |  | | | | | | |  | |
| Gelatinization temperature | | | GO:0005996 | | | | | | monosaccharide metabolic process | | | | | | |  | |
| Gelatinization temperature | | | GO:0006006 | | | | | | glucose metabolic process | | | | | | |  | |
| Gelatinization temperature | | | GO:0006007 | | | | | | glucose catabolic process | | | | | | |  | |
| Gelatinization temperature | | | GO:0006096 | | | | | | Glycolysis | | | | | | |  | |
| Gelatinization temperature | | | GO:0019320 | | | | | | hexose catabolic process | | | | | | |  | |
| Gelatinization temperature | | | GO:0046164 | | | | | | alcohol catabolic process | | | | | | |  | |
| Gelatinization temperature | | | GO:0046365 | | | | | | monosaccharide catabolic process | | | | | | |  | |
| *LOC_Os09g16000* | | *Broad-spectrum resistance to rice blast* | | | | | | | | | | | | | | |  |
| Blast disease resistance | | | NA | |  | | | | | | | |  |  | | |  |
| *LOC_Os09g26999* | | *Grain yield, panicle architecture* | | | | | | | | | | | |  | | |  |
| Spikelet number | | | NA | |  | | | | | | | |  |  | | |  |

^a^ For each trait found in literature with a fine-mapped candidate gene (‘Literature trait’ , ‘fine-mapped gene’), QTL traits in our dataset were obtained which were similar/related to the literature trait, and for which the fine-mapped gene occurred in one of the QTL regions (‘QTL compendium trait’). If this candidate gene was correctly prioritized by our approach, the biological processes involved are shown (‘BP’ , ‘BP Name’ ). NA, gene was not predicted as causal gene for the indicated trait.

^b^ LOC_Os08g07740 occurs as fine-mapped gene for two different traits.

**Table S4. Comparison with GWAS results^a^**

| Gene | Trait | Trait description |
| --- | --- | --- |
| LOC_Os03g22740 | TO:0000040 | Panicle length |
| LOC_Os10g39820 | TO:0000040 | Panicle length |
| LOC_Os10g40090 | TO:0000040 | Panicle length |
| LOC_Os03g27900 | TO:0000146 | Seed length |
| LOC_Os06g04310 | TO:0000196 | Amylose content |
| LOC_Os06g04330 | TO:0000196 | Amylose content |
| LOC_Os01g11940 | TO:0000207 | Plant height |
| LOC_Os01g11946 | TO:0000207 | Plant height |
| LOC_Os01g11952 | TO:0000207 | Plant height |
| LOC_Os01g12310 | TO:0000207 | Plant height |
| LOC_Os01g67770 | TO:0000207 | Plant height |
| LOC_Os02g31030 | TO:0000207 | Plant height |
| LOC_Os02g43300 | TO:0000207 | Plant height |
| LOC_Os02g56020 | TO:0000207 | Plant height |
| LOC_Os03g02290 | TO:0000207 | Plant height |
| LOC_Os03g05500 | TO:0000207 | Plant height |
| LOC_Os03g05720 | TO:0000207 | Plant height |
| LOC_Os03g05730 | TO:0000207 | Plant height |
| LOC_Os03g06070 | TO:0000207 | Plant height |
| LOC_Os05g32880 | TO:0000207 | Plant height |
| LOC_Os05g32890 | TO:0000207 | Plant height |
| LOC_Os05g32900 | TO:0000207 | Plant height |
| LOC_Os05g33030 | TO:0000207 | Plant height |
| LOC_Os05g33050 | TO:0000207 | Plant height |
| LOC_Os06g06440 | TO:0000207 | Plant height |
| LOC_Os06g09390 | TO:0000207 | Plant height |
| LOC_Os06g09570 | TO:0000207 | Plant height |
| LOC_Os06g09610 | TO:0000207 | Plant height |
| LOC_Os06g46270 | TO:0000207 | Plant height |
| LOC_Os06g46310 | TO:0000207 | Plant height |
| LOC_Os07g09740 | TO:0000207 | Plant height |
| LOC_Os07g42510 | TO:0000207 | Plant height |
| LOC_Os10g21560 | TO:0000207 | Plant height |
| LOC_Os10g21810 | TO:0000207 | Plant height |
| LOC_Os11g05470 | TO:0000207 | Plant height |
| LOC_Os02g50880 | TO:0000598 | Protein content |
| LOC_Os03g42020 | TO:0000734 | Grain length |

^a^Comparison between prioritized candidate genes and GWAS data indicated subset of genes which were associated with SNPs found by GWAS for relevant traits.

**Table S5. Genes in QTL regions for “heading date” annotated with** “regulation of flower development”

| **Gene** | **Existing annotation** |
| --- | --- |
| LOC_Os01g04750 | B3 DNA binding domain containing protein |
| LOC_Os01g04930 | MYB family transcription factor |
| LOC_Os01g68620 | signal peptide peptidase-like 2B |
| LOC_Os01g70920 | cullin-1, putative |
| LOC_Os01g74020 | MYB family transcription factor, putative |
| LOC_Os02g05510 | GATA transcription factor 25, putative |
| LOC_Os02g06910 | auxin response factor 6, putative |
| LOC_Os02g07430 | OsMADS29 - MADS-box family gene with MIKCc type-box |
| LOC_Os02g07770 | Myb transcription factor, putative |
| LOC_Os02g07930 | B-box zinc finger family protein, putative |
| LOC_Os02g12790 | GATA zinc finger domain containing protein |
| LOC_Os02g13130 | KH domain-containing protein, putative |
| LOC_Os02g13830 | osFTL13 FT-Like13 homologous to Flowering Locus T gene; contains Pfam profile PF01161: Phosphatidylethanolamine-binding protein |
| LOC_Os02g30700 | MYB family transcription factor, putative |
| LOC_Os02g31140 | major ampullate spidroin 2-2, putative |
| LOC_Os02g32950 | RCN2 Centroradialis-like1 homologous to TFL1 gene; contains Pfam profile PF01161: Phosphatidylethanolamine-binding protein |
| LOC_Os02g42950 | YABBY domain containing protein, putative |
| LOC_Os02g43150 | GATA zinc finger domain containing protein |
| LOC_Os02g43170 | B-box zinc finger family protein, putative |
| LOC_Os02g45080 | MYB family transcription factor, putative |
| LOC_Os02g56250 | GATA zinc finger domain containing protein |
| LOC_Os02g57530 | ethylene receptor, putative |
| LOC_Os02g57690 | kelch repeat protein, putative |
| LOC_Os02g57710 | signal peptide peptidase-like 2B, putative |
| LOC_Os03g01410 | Leucine Rich Repeat family protein |
| LOC_Os03g03070 | transcription factor, putative |
| LOC_Os03g03760 | MYB family transcription factor, putative |
| LOC_Os03g03850 | GATA zinc finger domain containing protein |
| LOC_Os03g06070 | la domain containing protein, putative |
| LOC_Os03g06654 | flavin monooxygenase, putative |
| LOC_Os03g54170 | OsMADS34 - MADS-box family gene with MIKCc type-box, |
| LOC_Os03g61570 | Protein |
| LOC_Os04g54400 | BTBN12 - Bric-a-Brac, Tramtrack, Broad Complex BTB domain with non-phototropic hypocotyl 3 NPH3 and coiled-coil domains, |
| LOC_Os04g54420 | protein of unknown function, DUF618 domain containing protein, |
| LOC_Os05g02300 | Core histone H2A/H2B/H3/H4 domain containing protein, putative |
| LOC_Os05g10580 | cullin family domain containing protein, putative |
| LOC_Os05g11380 | OsMADS66 - MADS-box family gene with MIKCc type-box |
| LOC_Os05g11414 | OsMADS58 - MADS-box family gene with MIKCc type-box |
| LOC_Os05g11510 | B-box zinc finger family protein, putative |
| LOC_Os05g32880 | response regulator receiver domain containing protein |
| LOC_Os05g32890 | response regulator receiver domain containing protein |
| LOC_Os05g33570 | pyruvate, phosphate dikinase, chloroplast precursor chloroplast precursor, putative |
| LOC_Os05g34110 | homeodomain-related, putative |
| LOC_Os05g34940 | OsMADS4 - MADS-box family gene with MIKCc type-box |
| LOC_Os06g06300 | osFTL3 FT-Like3 homologous to Flowering Locus T gene |
| LOC_Os06g06320 | osFTL2 FT-Like2 homologous to Flowering Locus T gene |
| LOC_Os06g06750 | OsMADS5 - MADS-box family gene with MIKCc type-box |
| LOC_Os06g11330 | OsMADS55 - MADS-box family gene with MIKCc type-box |
| LOC_Os06g11440 | transposon protein, putative, Mutator sub-class |
| LOC_Os06g44410 | histidine kinase, putative |
| LOC_Os06g44450 | CCT/B-box zinc finger protein, putative |
| LOC_Os06g45410 | MYB family transcription factor, putative |
| LOC_Os06g45650 | OsMADS30 - MADS-box family gene with MIKCc type-box |
| LOC_Os06g45900 | peptidyl-prolyl cis-trans isomerase-like 4, putative |
| LOC_Os06g46410 | auxin response factor, putative, LOC_Os06g46410.2 auxin response factor, putative |
| LOC_Os06g46880 | surp module family protein, putative |
| LOC_Os06g47150 | auxin response factor 18, putative |
| LOC_Os07g08520 | auxin response factor, putative |
| LOC_Os07g08540 | auxin response factor 20, putative |
| LOC_Os07g08600 | auxin response factor, putative |
| LOC_Os07g08880 | ES43 protein, putative |
| LOC_Os07g41370 | OsMADS18 - MADS-box family gene with MIKCc type-box |
| LOC_Os07g46180 | PWWP domain containing protein |
| LOC_Os07g47140 | CCT/B-box zinc finger protein, putative |
| LOC_Os07g48596 | Myb transcription factor, putative |
| LOC_Os08g06370 | MYB family transcription factor, putative |
| LOC_Os08g08830 | protein |
| LOC_Os08g32620 | ES43 protein, putative |
| LOC_Os08g33488 | OsMADS23 - MADS-box family gene with MIKCc type-box |
| LOC_Os08g34370 | SET domain containing protein |
| LOC_Os09g39270 | ZOS9-20 - C2H2 zinc finger protein |
| LOC_Os10g39130 | OsMADS56 - MADS-box family gene with MIKCc type-box |
| LOC_Os10g39550 | MYB family transcription factor, putative |
| LOC_Os10g40810 | GATA zinc finger domain containing protein, |
| LOC_Os12g06850 | transcription elongation factor protein, putative |
| LOC_Os12g07120 | GATA zinc finger domain containing protein |
| LOC_Os12g42310 | serine/threonine-protein phosphatase BSL2, putative |
| LOC_Os12g42610 | YABBY domain containing protein, putative |
| LOC_Os12g42970 | GATA zinc finger domain containing protein |

**Table S6. Genes in QTL regions for “heading date” annotated with “ribonucleoprotein complex biogenesis” and “ribosome biogenesis”**

| **Gene** | **Existing annotation** |
| --- | --- |

| LOC_Os01g04730 | ribosomal protein L24, putative, expressed |
| --- | --- |
| LOC_Os01g07080 | DEAD-box ATP-dependent RNA helicase, putative |
| LOC_Os01g68320 | DEAD-box ATP-dependent RNA helicase 30, putative |
| LOC_Os01g69970 | WD domain, G-beta repeat domain containing protein, expressed |
| LOC_Os01g70010 | ribosomal protein L7Ae, putative, expressed |
| LOC_Os01g73900 | DEAD-box ATP-dependent RNA helicase, putative, expressed |
| LOC_Os02g05660 | DEAD-box ATP-dependent RNA helicase 35A, putative, expressed |
| LOC_Os02g06500 | DSHCT domain containing protein, expressed |
| LOC_Os02g12840 | DEAD-box ATP-dependent RNA helicase, putative, expressed |
| LOC_Os02g30624 | LSM domain containing protein, expressed |
| LOC_Os02g30840 | RNA pseudouridine synthase, putative, expressed |
| LOC_Os02g32760 | 60S acidic ribosomal protein, putative, expressed |
| LOC_Os02g42406 | DEAD-box ATP-dependent RNA helicase, putative, expressed |
| LOC_Os02g42860 | DEAD-box ATP-dependent RNA helicase, putative, expressed |
| LOC_Os02g44810 | tRNA pseudouridine synthase family protein, putative, expressed |
| LOC_Os02g55260 | DEAD-box ATP-dependent RNA helicase, putative, expressed |
| LOC_Os02g56020 | methyltransferase, putative, expressed |
| LOC_Os02g57590 | rRNA 2-O-methyltransferase fibrillarin 2, putative, expressed |
| LOC_Os02g57980 | DEAD/DEAH box helicase, putative, expressed |
| LOC_Os03g01830 | DEAD-box ATP-dependent RNA helicase, putative, expressed |
| LOC_Os03g05980 | 40S ribosomal protein S9-2, putative, expressed |
| LOC_Os03g06220 | DEAD-box ATP-dependent RNA helicase, putative, expressed |
| LOC_Os03g18410 | DNA-directed RNA polymerase subunit, putative, expressed |
| LOC_Os03g52470 | WD domain, G-beta repeat domain containing protein, expressed |
| LOC_Os03g55150 | eukaryotic translation initiation factor 5A, putative, expressed |
| LOC_Os03g60930 | RNA recognition motif containing protein, putative, expressed |
| LOC_Os03g61030 | transcription termination factor nusG family protein, expressed |
| LOC_Os03g61220 | DEAD-box ATP-dependent RNA helicase 3, putative, expressed |
| LOC_Os03g61560 | expressed protein |
| LOC_Os05g01990 | DEAD-box ATP-dependent RNA helicase, putative, expressed |
| LOC_Os05g02400 | RNA recognition motif containing protein, expressed |
| LOC_Os05g11710 | ribosomal protein L5, putative, expressed |
| LOC_Os05g35540 | GTPase of unknown function domain containing protein, putative, expressed |
| LOC_Os06g12910 | XPA-binding protein 2, putative, expressed |
| LOC_Os06g16290 | ribosomal protein L7Ae, putative, expressed |
| LOC_Os06g46930 | ribosomal protein L24, putative, expressed |
| LOC_Os07g05580 | ribosomal protein L7Ae, putative, expressed |
| LOC_Os07g08330 | ribosomal protein L4, putative, expressed |
| LOC_Os07g10250 | DEAD-box ATP-dependent RNA helicase 52B, putative, expressed |
| LOC_Os07g10350 | S1 RNA binding domain containing protein, expressed |
| LOC_Os07g10720 | 40S ribosomal protein S15a, putative, expressed |
| LOC_Os07g41190 | WD domain, G-beta repeat domain containing protein, expressed |
| LOC_Os07g41790 | LSM domain containing protein, expressed |
| LOC_Os07g43470 | GTP-binding protein, putative, expressed |
| LOC_Os07g43510 | 40S ribosomal protein S9, putative, expressed |
| LOC_Os07g43980 | DEAD-box ATP-dependent RNA helicase, putative, expressed |
| LOC_Os07g44190 | h/ACA ribonucleoprotein complex subunit 4, putative, expressed |
| LOC_Os07g44230 | ribosomal protein L7Ae/L30e/S12e/Gadd45 family protein, putative, expressed |
| LOC_Os07g44970 | XPA-binding protein 2, putative, expressed |
| LOC_Os07g45360 | DEAD/DEAH box helicase domain containing protein, expressed |
| LOC_Os07g46440 | ribosomal protein, putative, expressed |
| LOC_Os07g46760 | RNA recognition motif containing protein, putative, expressed |
| LOC_Os07g47300 | spo0B-associated GTP-binding protein, putative, expressed |
| LOC_Os07g47420 | 60S ribosome subunit biogenesis protein NIP7, putative, expressed |
| LOC_Os07g48300 | eukaryotic translation initiation factor 2 subunit beta, putative, expressed |
| LOC_Os07g49210 | helicase conserved C-terminal domain containing protein, expressed |
| LOC_Os08g06230 | nucleolar GTP-binding protein 1, putative, expressed |
| LOC_Os08g06344 | transposon protein, putative, CACTA, En/Spm sub-class, expressed |
| LOC_Os08g08040 | LSM domain containing protein, expressed |
| LOC_Os08g31810 | RNA recognition motif containing protein, putative, expressed |
| LOC_Os08g32090 | DEAD-box ATP-dependent RNA helicase, putative, expressed |
| LOC_Os10g25110 | pumilio-family RNA binding repeat containing protein, expressed |
| LOC_Os10g39820 | zinc knuckle domain containing protein, expressed |
| LOC_Os10g42320 | nonsense-mediated mRNA decay protein 3, putative, expressed |
| LOC_Os12g03090 | ribosomal protein, putative, expressed |
| LOC_Os12g03822 | WD domain, G-beta repeat domain containing protein, expressed |
| LOC_Os12g03880 | 60S acidic ribosomal protein P0, putative, expressed |
| LOC_Os12g04010 | M-phase phosphoprotein 10, putative, expressed |
| LOC_Os12g04160 | ribosomal protein L24, putative, expressed |
| LOC_Os12g07010 | ribosomal protein L3, putative, expressed |
| LOC_Os12g07300 | N-acetyltransferase 10, putative, expressed |
| LOC_Os12g42370 | GTPase of unknown function domain containing protein, putative, expressed |

**References**

1. Ashikari, M., Sakakibara, H., Lin, S., Yamamoto, T., Takashi, T., Nishimura, A., Angeles, E.R., Qian, Q., Kitano, H. and Matsuoka, M. (2005) Cytokinin oxidase regulates rice grain production. *Science*, **309**, 741-745.

2. Wang, P., Zhou, G., Yu, H. and Yu, S. (2011) Fine mapping a major QTL for flag leaf size and yield-related traits in rice. *TAG. Theoretical and applied genetics. Theoretische und angewandte Genetik*, **123**, 1319-1330.

3. Liu, T., Mao, D., Zhang, S., Xu, C. and Xing, Y. (2009) Fine mapping SPP1, a QTL controlling the number of spikelets per panicle, to a BAC clone in rice (Oryza sativa). *TAG. Theoretical and applied genetics. Theoretische und angewandte Genetik*, **118**, 1509-1517.

4. Abe, A., Takagi, H., Fujibe, T., Aya, K., Kojima, M., Sakakibara, H., Uemura, A., Matsuoka, M. and Terauchi, R. (2012) OsGA20ox1, a candidate gene for a major QTL controlling seedling vigor in rice. *TAG. Theoretical and applied genetics. Theoretische und angewandte Genetik*, **125**, 647-657.

5. Wang, E., Wang, J., Zhu, X., Hao, W., Wang, L., Li, Q., Zhang, L., He, W., Lu, B., Lin, H. *et al.* (2008) Control of rice grain-filling and yield by a gene with a potential signature of domestication. *Nature genetics*, **40**, 1370-1374.

6. Su, Y., Rao, Y., Hu, S., Yang, Y., Gao, Z., Zhang, G., Liu, J., Hu, J., Yan, M., Dong, G. *et al.* (2011) Map-based cloning proves qGC-6, a major QTL for gel consistency of japonica/indica cross, responds by Waxy in rice (Oryza sativa L.). *TAG. Theoretical and applied genetics. Theoretische und angewandte Genetik*, **123**, 859-867.

7. Shan, J.X., Zhu, M.Z., Shi, M., Gao, J.P. and Lin, H.X. (2009) Fine mapping and candidate gene analysis of spd6, responsible for small panicle and dwarfness in wild rice (Oryza rufipogon Griff.). *TAG. Theoretical and applied genetics. Theoretische und angewandte Genetik*, **119**, 827-836.

8. Gao, Z., Zeng, D., Cheng, F., Tian, Z., Guo, L., Su, Y., Yan, M., Jiang, H., Dong, G., Huang, Y. *et al.* (2011) ALK, the key gene for gelatinization temperature, is a modifier gene for gel consistency in rice. *Journal of integrative plant biology*, **53**, 756-765.

9. Ookawa, T., Hobo, T., Yano, M., Murata, K., Ando, T., Miura, H., Asano, K., Ochiai, Y., Ikeda, M., Nishitani, R. *et al.* (2010) New approach for rice improvement using a pleiotropic QTL gene for lodging resistance and yield. *Nature communications*, **1**, 132.

10. Dai, X., Ding, Y., Tan, L., Fu, Y., Liu, F., Zhu, Z., Sun, X., Sun, X., Gu, P., Cai, H. *et al.* (2012) LHD1, an allele of DTH8/Ghd8, controls late heading date in common wild rice (Oryza rufipogon). *Journal of integrative plant biology*, **54**, 790-799.

11. Wei, X., Xu, J., Guo, H., Jiang, L., Chen, S., Yu, C., Zhou, Z., Hu, P., Zhai, H. and Wan, J. (2010) DTH8 suppresses flowering in rice, influencing plant height and yield potential simultaneously. *Plant physiology*, **153**, 1747-1758.

12. Wang, S., Wu, K., Yuan, Q., Liu, X., Liu, Z., Lin, X., Zeng, R., Zhu, H., Dong, G., Qian, Q. *et al.* (2012) Control of grain size, shape and quality by OsSPL16 in rice. *Nature genetics*, **44**, 950-954.

13. Li, J., Zhang, W., Wu, H., Guo, T., Liu, X., Wan, X., Jin, J., Hanh, T.T., Thoa, N.T., Chen, M. *et al.* (2011) Fine mapping of stable QTLs related to eating quality in rice (Oryza sativa L.) by CSSLs harboring small target chromosomal segments. *Breeding science*, **61**, 338-346.

14. Liu, Y., Liu, B., Zhu, X., Yang, J., Bordeos, A., Wang, G., Leach, J.E. and Leung, H. (2013) Fine-mapping and molecular marker development for Pi56(t), a NBS-LRR gene conferring broad-spectrum resistance to Magnaporthe oryzae in rice. *TAG. Theoretical and applied genetics. Theoretische und angewandte Genetik*, **126**, 985-998.

15. Huang, X., Qian, Q., Liu, Z., Sun, H., He, S., Luo, D., Xia, G., Chu, C., Li, J. and Fu, X. (2009) Natural variation at the DEP1 locus enhances grain yield in rice. *Nature genetics*, **41**, 494-497.
